# From In Silico to a Cellular Model: Molecular Docking Approach to Evaluate Antioxidant Bioactive Peptides

**DOI:** 10.3390/antiox12030665

**Published:** 2023-03-08

**Authors:** Federica Tonolo, Alessandro Grinzato, Alberto Bindoli, Maria Pia Rigobello

**Affiliations:** 1 Department of Biomedical Sciences, University of Padova, Via U. Bassi 58/b, 35131 Padova, Italy; 2 Department of Comparative Biomedicine and Food Science, University of Padova, Viale dell’Università, 35020 Padova, Italy; 3 European Synchrotron Radiation Facility, 71 Avenue des Martyrs, 38000 Grenoble, France; 4 Institute of Neuroscience (CNR), Viale G. Colombo 3, 35131 Padova, Italy

**Keywords:** bioactive peptides, molecular docking, molecular mechanism, antioxidant, Keap1/Nrf2

## Abstract

The increasing need to counteract the redox imbalance in chronic diseases leads to focusing research on compounds with antioxidant activity. Among natural molecules with health-promoting effects on many body functions, bioactive peptides are gaining interest. They are protein fragments of 2–20 amino acids that can be released by various mechanisms, such as gastrointestinal digestion, food processing and microbial fermentation. Recent studies report the effects of bioactive peptides in the cellular environment, and there is evidence that these compounds can exert their action by modulating specific pathways. This review focuses on the newest approaches to the structure–function correlation of the antioxidant bioactive peptides, considering their molecular mechanism, by evaluating the activation of specific signaling pathways that are linked to antioxidant systems. The correlation between the results of in silico molecular docking analysis and the effects in a cellular model was highlighted. This knowledge is fundamental in order to propose the use of bioactive peptides as ingredients in functional foods or nutraceuticals.

## 1. Introduction

During the last few decades, demographic changes due to better living conditions have led to a growing increase in the population of people older than 50 years, which is estimated to be more than one-third of the European population [1]. In particular in Europe and Northern America, one in four people are aged 65 or over and the number of people older than 80 years is expected to triple by 2050 [1]. In this scenario, chronic and age-related diseases, such as diabetes, cardiovascular disease and obesity are gaining increasing relevance, as an inflammatory process and an imbalance of cellular redox status characterize these pathologies. For this reason, scientists and industries are focusing their attention on new strategies to cope with disease occurrence.

What does an antioxidant molecule mean now? A new approach regards nutraceuticals, i.e., food-derived bioactive molecules that promote health benefits beyond their basic nutritional values. Bioactive molecules are known for their antioxidant and anti-inflammatory properties and they are present in some fruits, vegetables and herbal plants such as phenolic acids, anthocyanins, polyphenols and flavonoids. In recent years, the category of antioxidant bioactive peptides has gained increasing interest.

**Figure 1:**
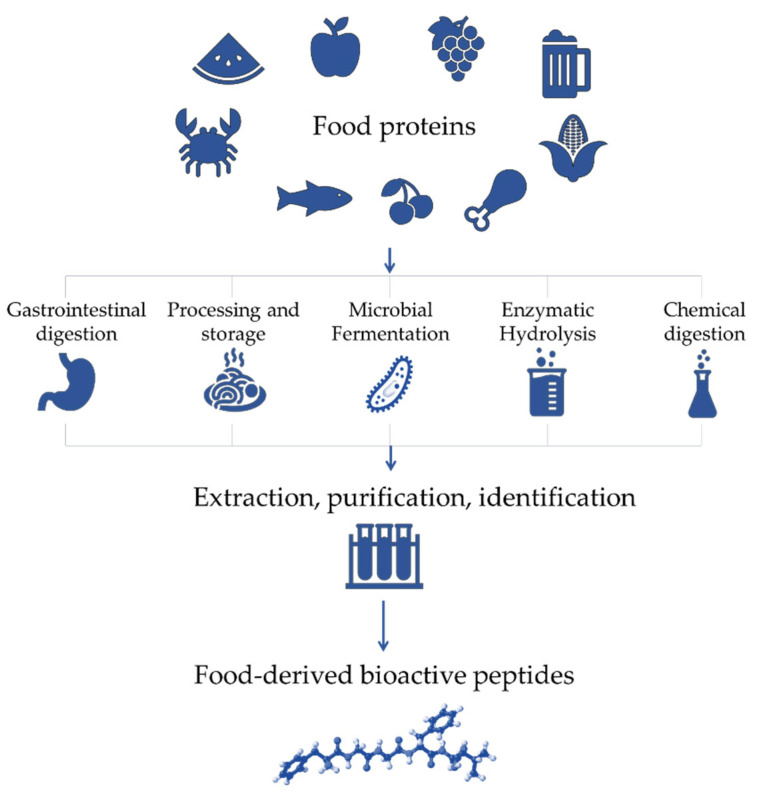
Production steps of bioactive peptides from food proteins.

The health-promoting roles of naturally occurring dietary proteins are due to the presence of specific peptide sequences that are encrypted in inactive forms inside the parent protein. These molecules, called bioactive peptides, can be released by various mechanisms, such as gastrointestinal digestion, food processing, fermentation, protein enzymatic hydrolysis and chemical digestion (Figure 1) [2,3,4].

Bioactive peptides are obtained from different food matrices, originating from animal and plant proteins, for example egg, milk, meat, soy, seafood and plants [5]. The various sources and production mechanisms have been exhaustively examined in recent review articles [6,7]. Moreover, although the structure–function relationships of bioactive peptides are not well-established, many of them share some common properties. For instance, peptides contain 2 to 20 amino acids and are generally rich in hydrophobic residues [8]. These molecules exert a positive impact on human health and beneficial effects on many bodily functions, such as antimicrobial, antithrombotic, immunomodulatory, anti-hypertensive, opioid, anti-inflammatory and antioxidant activities [4,9,10,11,12,13,14,15,16,17,18]. Of note, for example, VPP and IPP are two tripeptides derived from milk caseins that have been shown to reduce high blood pressure in humans and to be vascular protective, antioxidant and able to decrease inflammation effects [10,12]. In particular, VPP tripeptide can control the interactions between the leukocyte and endothelial cell in vitro, inhibiting the pro-inflammatory c-Jun N-terminal kinase pathway [19]. To counteract the inflammatory process, bioactive peptides can modulate the activation of immune cells or various signaling pathways, such as the nuclear factor kappa-light-chain-enhancer of the activated B-cell (NF-κB) system [17,18,19]. For example, egg-, soy- and dairy-derived peptides, such as IRW, IQW, DQWL and lunasin, are able to downregulate, through the modulation of the NF-κB pathway, the cytokine-induced inflammatory protein expression in different cell types in vitro, such as HUVECs, RAW 264.7 and Caco-2 cells [18,19]. It is well-known that the different effects of the bioactive peptides are controlled by their amino acid sequence, as they could interact with the proteins that are present in the cell environment and modulate their physiological processes [20]. These different activities are related to the specific sequence and structure of the peptides. For example, the amino acids Pro, His, Trp, Phe, Leu and Tyr are more frequent in bioactive peptides with antidiabetic, antihypertensive and antioxidant capacities [21]. Nevertheless, from a structural point of view, there is no consensus on the architecture of bioactive peptides [20,22]. Thus, the use of bioactive peptides has gained much interest, but knowledge of their molecular mechanisms is still little explored. In fact, the current research on bioactive peptides is mainly carried out through in vitro tests and there are recent evidences that bioactive peptides can exert their action by modulating specific molecular pathways [5,23]. These compounds are very promising for the fallouts on nutraceuticals and functional foods as well as in drug development [6,7,24,25,26,27]. Therefore, understanding the molecular basis that is involved in the activation of the signaling systems and is stimulated by antioxidant food-derived bioactive peptides is a key point to propose these molecules as nutraceuticals or functional food ingredients. For this reason, the main goal of this review is to put together the newest results and strategies about the structure–function correlation of the antioxidant bioactive peptides considering their molecular mechanisms in cellular models. In the literature, the mechanisms of action exerting antioxidant effects by bioactive peptides can also be attributed to different signaling pathways [28,29]. Here, we restrict our attention to bioactive peptides that can directly interact with Keap1 activating the Keap1/Nrf2 pathway.

## 2. Antioxidant Bioactive Peptides

In the pathogenesis of chronic diseases, in addition to inflammatory processes, an imbalance of the redox status also occurs. The need to counteract this stress condition prompted us to focus on antioxidant peptides, which are the main subject of this research.

Once released, antioxidant bioactive peptides show an antioxidant activity higher than the native proteins and can exert their action through various mechanisms. The first one is the classic way of action of antioxidants, in which peptides act as direct scavengers of free radicals [5,30]. These compounds donate a hydrogen atom to a free radical that is harmful for the cell and, in addition to the neutralization of the potentially toxic-free radical, the product of the reaction is a resonance-stabilized radical form of the antioxidant peptide, which is scarcely reactive and may be regenerated to the native form. Moreover, the antioxidant peptides can also act as metal chelators, thanks to the presence of specific amino acids in their sequence, such as histidine [5,30]. Other peptides are able to interact with key molecules of signaling pathways that are capable of regulating specific genes that lead to the expression of proteins involved in the modulation of the antioxidant response, such as the Kelch-like ECH-associated protein 1/nuclear factor erythroid 2-related factor 2 pathway (Keap1/Nrf2) [3,5].

Although the relationship between their structure and activity has not been established yet, antioxidant peptides share common features. In general, they consist of small protein fragments with a low molecular weight and are characterized by a high frequency of Pro, His, Tyr, Trp, Arg and Met in their sequences [16,25,31]. The physicochemical properties of these bioactive peptides directly influence their antioxidant activity. Especially the presence of amino acids with an aromatic ring, such as Tyr, Trp and Phe, which promote the antioxidant capacity due to the formation of the resonance-stabilized radical form of the peptides [16,25,30]. Moreover, hydrophobic amino acids enhance the capability of the peptides to enter the cells and also to reach the mitochondria, one of the major sites of free radical production [25,30]. The hydrophobicity is an important characteristic of the bioactive peptide’s antioxidant activity as it helps protect PUFAs and other lipophilic targets from oxidation [25,30]. Furthermore, the presence of histidine increases the capacity of the peptides to chelate metals and to quench lipid and hydroxyl radicals. Their antioxidant effectiveness rises in the presence of a hydrophobic amino acid at the N-terminus and a charged amino acid at the C-terminus [4,25,30].

### 2.1. Applications of Antioxidant Bioactive Peptides

The main application of antioxidant bioactive peptides is their inclusion in the composition of nutraceuticals, functional foods and supplemented foods, or they could be used as drugs [4,16,32,33]. Moreover, milk-derived bioactive peptides can be employed as nutrient supplements in infant formula products and baby foods [34]. However, a wide knowledge on the molecular mechanisms, toxicity and bioavailability is required. Further studies are needed to evaluate the physiological role and efficacy of these molecules as there is a lack of information about these compounds in vivo and particularly in human clinical studies. Additionally, the industrial production of bioactive peptides needs to be better developed in order to be more affordable and consistent so to obtain effective products that are able to improve human health [8,27].

Antioxidant bioactive peptides are gaining interest in addition to their health-promoting action, because they could replace, in adequate concentrations, synthetic antioxidant compounds such as butylated hydroxytoluene (BHT) and butylated hydroxyanisole (BHA) [5,23,31].

### 2.2. Redox Signalling

In physiological conditions, ROS, RNS, oxidized proteins and lipid peroxidation reaction products formed in cells can interact with specific proteins causing post-translational modifications that can transmit and amplify specific signals. In this process, defined as redox signaling, many transcription factors, kinases, receptors and channels are involved. They are able to act as the sensors of changes in the redox status of the cell and modify their specific conformations [35].

**Figure 2:**
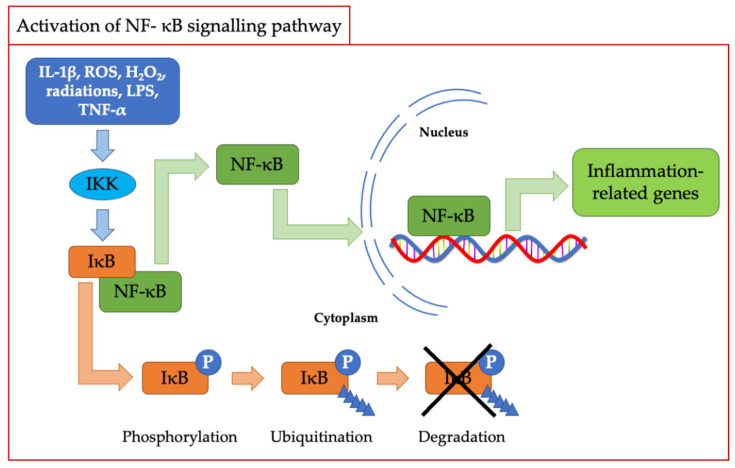
Scheme of NF-kB signaling pathway activation.

As described above, oxidative stress and inflammation are strictly correlated and probably for this reason, the first mammalian transcription factor shown to be regulated by oxidation was NF-κB [36]. The latter plays a crucial role in mediating the inflammatory response and is regulated by various mediators, including ROS and in particular H_2_O_2_ [37]. In this case, as depicted in Figure 2, the activation of NF-κB is mediated by the classic IKK-dependent pathway; then, the inhibitory protein IκB is phosphorylated and subsequently eliminated by the proteasome. This process promotes the migration of NF-κB from the cytosol to the nucleus, leading to the transcription of downstream-regulated genes [35,38].

**Figure 3:**
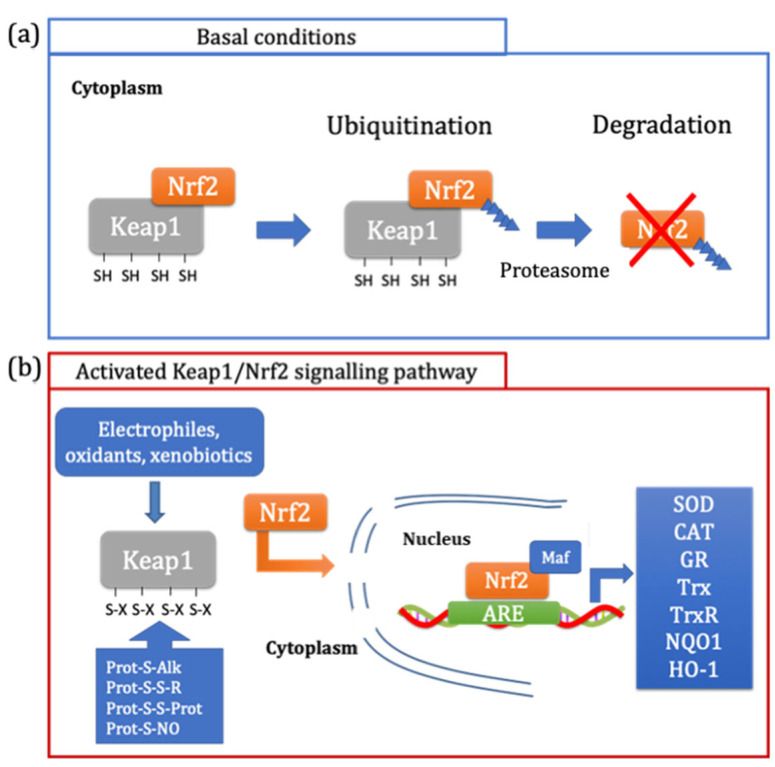
Regulation of Keap1/Nrf2 pathway. In basal conditions, Keap1 leads to the ubiquitination of Nrf2, promoting its degradation through its translocation to the proteasome (**a**). The Keap1/Nrf2 pathway is activated by electrophiles, oxidants and xenobiotics, which cause the oxidation of specific SH groups of Keap1. This modification causes the dissociation of Nrf2, which moves from the cytosol to the nucleus where it can bind, in association with the Maf protein, to the antioxidant response elements (ARE), leading to the antioxidant and phase II enzymes expression (**b**).

The major pathway involved in the maintenance of redox homeostasis is the Keap1/Nrf2-ARE pathway [39,40]. In particular, the Kelch-like ECH-associated protein 1 (Keap1) is the repressor protein of the nuclear factor erythroid 2-related factor 2 (Nrf2). In basal conditions, Keap1 binds Nrf2 and promotes its degradation via the ubiquitin proteasome pathway (Figure 3a) [40]. The amino acid residues of Keap1 involved in the binding with Nrf2 are contained in the Kelch repeats sequence (aa 327–609), where Arg380 and Arg415 play a crucial role in Nrf2 binding [40]. Nrf2 interacts with Keap1 through the Neh1 domain, which contains two highly conserved amino acid motifs, DLG (aa 29–31) and ETGE (aa 79–82) [39,41]. Moreover, Keap1 is a cysteine-rich protein, making it an ideal redox sensor [40]. Some residues (Cys273 and Cys278) cover a critical role by inducing the change in conformation that is stimulated by oxidants and electrophiles (Figure 3b) [40]. Once Keap1 is activated, the interaction between the two proteins no longer occurs and at the same time, Nrf2 is not ubiquitinated and degraded; therefore, the newly synthetized Nrf2 accumulates in the cytosol [41]. Nrf2 is a transcription factor that, when the dissociation from its repressor (Keap1) occurs, moves from the cytosol to the nucleus [39]. Here, Nrf2, together with small Maf proteins, binds the antioxidant response element (ARE) in the regulatory regions of the antioxidant and phase II enzymes, such as superoxide dismutase (SOD), catalase (CAT), glutathione reductase (GR), thioredoxin (Trx), thioredoxin reductase (TrxR), NAD(P)H quinone oxidoreductase 1 (NQO1) and heme oxygenase (HO-1), and promotes the transcription of the genes involved in cell detoxification from oxidants and xenobiotics [39].

Many are the activators of the Keap1/Nrf2 pathway, including a large number of endogenous molecules, such as lipid oxidation products and ROS, in addition to exogenous compounds of natural or synthetic origins with different and chemically unrelated structures, such as selenium compounds and heavy metals [41]. On the other hand, other compounds such as isothiocyanates, polyphenols and carotenoids, can activate this signaling pathway [40,41]. These molecules can modify the sensitive cysteine of Keap1 either indirectly, by generating oxidant compounds through autoxidation, or with a direct effect, for example by an alkylating reaction [35,42].

## 3. In Silico Prediction of Bioactive Peptides Docking

As documented in numerous studies, virtual screening, particularly molecular docking, is increasingly used to evaluate the possible interaction of bioactive peptides. The high likelihood of success, quick identification of positives and cost reduction compared to standard techniques such as high-throughput screening may fuel molecular docking’s rising popularity. In virtual screening, a database of peptides is analyzed against a target of interest, and a sub-group is identified and recommended for in vitro testing [7].

Various in silico methods could be applied to predict the protein–peptide interaction by investigating parameters such as the binding affinity, conformational stability, hydrophobicity and a list of active residues involved in the binding. Moreover, some methods utilize the present data in the bioactive chemical databases, examining the overall chemical environment and comparing the peptide behavior with those of already well-known pharmaceuticals [43,44]. Further, the recent use of artificial intelligence (AI) improved the description of the bioactivity profile of selected targets, thanks to the use of multivariate statistics that take into account a plethora of interaction variables and chemical properties [45,46].

Among the numerous in silico techniques described, molecular docking analysis is one of the most frequently used tools in drug design research and virtual screening studies to discover novel active molecules that are derived from natural sources. This biomolecular simulation is used to predict binding sites to elucidate the mechanism of molecular recognition by simulating the spontaneous binding process of biomolecules, and to explore the relationship between molecular recognition and molecular structure [46].

Molecular docking involves the fitting of a peptide or a ligand into a target’s active site while an associated algorithm forecasts its binding affinity [47]. The binding site generates a variety of poses; the scoring function that is linked with it ranks the best binding poses and separates the binders from decoys [48].

The three strategies available for peptide–protein docking are template-based, local and global docking. These three methods have different prediction accuracies, mostly related to the exactness of the input information [49].

Template-based docking creates a model starting from known structures and can be advantageous in terms of its computational cost and quality of the prediction, but only if the template is near the complex under investigation [50,51,52,53].

Local docking techniques seek a peptide-binding pose near a user-specified binding location. As a result, the data input influences of the precision of the docking. The better the results, the more precisely the binding location is characterized [49].

Global docking scans the target structure to find all the possible binding poses. The most fundamental step in this approach is to perform thorough rigid-body docking while treating the protein and peptide input conformations as rigid. A more challenging use of this method is to predict the peptide conformation from a user-provided sequence. This process typically consists of three steps:

1. The input peptide conformations should be created by threading the sequence onto a predefined set of template conformations or simulating peptide folding in a solution [54,55].
2. Rigid-body docking [49].
3. Scoring and model refinement [49].

Independent of the strategies used, there are at least three critical factors to consider while performing a peptide docking prediction or evaluating the results.

Firstly, the so-called flexibility problem, as no significant conformational changes could be predicted during a rigid-body docking. Therefore, a limited sampling of both the ligand and receptor conformations in a pose prediction could be analyzed during the simulation.

In molecular docking, the protein structures are treated as static receptors, which are unable to react to ligand perturbations or exogenous molecules (such as ions and solvents) and surroundings (e.g., pH and electric field) [56]. While in their natural environment, the targeting proteins are slightly flexible due to the continual motion between various conformational states with similar energies and they can adopt a variety of conformations depending on the ligand to which they bind [57]. Solvation effects and entropy changes are two examples of intermolecular interactions that cannot be reliably predicted [57,58].

Secondly, the so-called scoring problem involves selecting the complex with the highest affinity and prediction accuracy from many predicted models.

Lastly, it is worth being reminded that the prediction accuracy is strongly related to the accuracy of the starting model, and, therefore, it somehow depends on the quality and quantity of the structural data of the target available.

In addition, it is also important to mention that no computational prediction experiment is considered complete without an extensive integration and confrontation between the predicted and the experimental findings [59].

### In Silico Prediction of Antioxidant Peptides Activity

Nowadays, a large number of papers have clarified by molecular docking the mechanisms of action of several food-derived peptides that showed antioxidant activity (Table 1 and Table S1). Among all the possible target proteins, Keap1 appears to be the most frequently used in docking analyses of bioactive peptides such as the Nrf2/Keap1 pathway, which is particularly relevant when cells are under oxidative stress [60,61,62,63]. Moreover, one of the biggest limitations of molecular docking is the number of structural data of the target available, which is definitely not a restriction for Keap1. It is intriguing that, up to now, more than 150 Keap1 structures were deposited in the PDB database, including the apo- and inhibitor-bound versions. Additionally, Keap1 overcomes the inability of molecular docking to leverage the receptor’s flexibility, such as the Kelch domain surface, which shows exceptional rigidity with only the arginine residues implicated in peptide binding, exhibiting notable conformational changes between the apo- and peptide-bound crystal structures, as it is possible to appreciate it superimposing the apo- and the peptide-bound X-ray crystallographic structures [64,65]. In addition, Keap1 has many other properties that facilitate docking predictions.

(i) Both the structure of the human and mouse Keap1-Kelch domain have been resolved at a high-resolution using X-ray crystallography. The in-depth analysis of these structures demonstrates that the essential interactions between the binding partners are conserved across species.

(ii) The Keap1-Kelch domain has a unique binding cavity with polar structures and a size that peptides, but also small molecules, can address, in contrast to typical protein–protein interaction interfaces that are generally very flat and primarily hydrophobic.

(iii) According to the crystal structures, there are no conserved and bounded water molecules. Therefore, no water molecules replacement or interactions should be considered during the simulations.

(iv) Studies on mutations and pharmacological effects demonstrate that inhibiting the low-affinity interaction between the DLG motif of Nrf2 and Keap1 is already sufficient to prevent Nrf2 from being ubiquitinated and to activate the transcription factor [66].

**Table 1:** Bioactive peptides that interact with Kelch domain and the validation of their effects in cellular studies.

aa	Sequence	pI	Net Charge	Kelch Domain Interaction	Validation in Cellular Studies	Ref.	Ref.
8	KVLPVPEK	9.63	+1	Gln337, Ser383, Asn382, Asn387, Tyr334, Arg380 and Ser363	yes	[60]
5	EDYGA	2.87	−3	Arg415	no	[61]
10	DEQIPSHPPR	5.21	−1	Arg380, Asn382 and Arg415	yes	[62]
10	SLVNNDDRDS	3.53	−2	Tyr334, Arg380, Asn414, Arg415 and Tyr525	yes	[62]
11	VNPESQQGSPR	6.99	0	Tyr334, Arg415, Arg483 and Tyr572	yes	[62]
11	IGINAENNQRN	6.99	0	Ser363, Asn382, Asp385, Arg415, Arg483, Ser508, Gln530 and Ser602	yes	[62]
12	FVDAQPQQKEEG	3.77	−2	Tyr334, Arg380, Asn382, Asn387, Arg415, Arg483 and Gln530	yes	[62]
12	FGREEGQQQGEE	3.7	−3	Arg336, Ser363, Arg380, Asn382, Arg415, Arg483, Tyr525, Tyr572 and Ser602	yes	[62]
13	MRKPQQEEDDDDE	3.53	−5	Arg380, Asp389, Arg415, Ser431, His436, Arg483 and Ser602	yes	[62]
9	YLAGNQEQE	3.09	−2	Arg380 and Arg415	yes	[62]
14	NALEPDHRVESEGG	4.18	−3	Tyr334, Arg380, Asn414, Arg415, His432 and Ser602	yes	[62]
14	KEQQQEQQQEEQPL	3.70	-3	Tyr334, Arg380, Asn414, Arg415, Ser431, Arg483 and Ser602	yes	[62]
14	HEQKEEHEWHRKEE	5.31	−3	Arg336, Ser363, Arg380, Asn382, Asp385, Asn387, Asp389, Asn414, Arg415, Arg483 and Ser602	yes	[62]
14	GKHQQEEENEGGSI	4.28	−3	Ser363, Arg380, Asp389, Asn414, Tyr572 and Ser602	yes	[62]
14	QGPIVLNPWDQVKR	10.12	1	Arg380, Asn382, Asn387, Arg415, His432, Ser508, Tyr525, Gln530, His575 and Thr576	yes	[63]
15	NTVPAKSCQAQPTTM	8.97	1	Arg336, Arg380, Asn382, Arg415, Gly433, Ile435, Gly509, Tyr572, Thr576 and Ser602	yes	[63]
17	APSFSDIPNPIGSENSE	2.93	−3	Arg336, Ser363, Arg415 and Tyr572	yes	[63]
9	VLSTSFCPK	8.67	+1	Cys434, Asp479, Thr458, Leu457, Met499, Cys489, Glu542, Arg459, Met499, Arg498 and Glu542	yes	[67]
9	VLSTSFYPK	9.48	+1	Cys434, Asp459, Met499, Cys489, Glu542, His436, Gly480, Arg459 and Thr458	yes	[67]
8	IVLPDEGK	1.01	−1	Arg380 and Arg415, His436, Ile461, Arg483, Ser508, Ser555 and Tyr572	yes	[68]
10	SDGSNIHFPN	4.98	−1	Leu365, Arg380 and Arg415. Additionally, Gly462, Arg483, Ala510, Tyr525, Ala556, Leu557, Tyr572 and Gly603	yes	[68]
17	PGMLGGSPPGLLGGSPP	5.25	0	Gly364, Leu365, Ala366 and Arg380, Asn382, Arg415, Ile416, Gly433, Arg483, Cys434, Ala510, Tyr525, Leu557, Tyr572, Gly603 and Val604	yes	[68]
6	VLFSNY	5.53	0	Arg380, Asn382, Arg415, Arg483, Ser508, Ser555 and Ser602	yes	[69]
7	FYSLHTF	7.64	0	Arg380, Asn414, Arg415, Ser431, Gln530 and Ser602	yes	[69]
7	VYGYADK	6.41	0	Arg336, Arg380, Asn414, Arg415, Gln530 and Ser602	yes	[69]
8	TFQGPPHG	7.91	0	Arg380, Asn382, Asn414, Arg415, Ser431, Gly433, Ser555 and Ser602	yes	[69]
8	YTPEYQTK	6.5	0	Tyr334, Ser363, Arg380, Asn382, Arg415, Ser431, His436, Arg483, Tyr525, Gln530, Ser555 and Ser602	yes	[69]
10	SSGHTLPAGV	7.89	0	Arg380, Arg415, Arg483, Tyr525, Gln530 and Ser555	yes	[69]
10	SGDWSDIGGR	3.92	−1	Tyr334, Gly364, Arg380, Arg483, Tyr525, Gln530, gly574 and Ser602	yes	[70]
6	RDPEER	4.32	−1	Asn382, Arg380 and Tyr334	no	[71]
5	SPSSS	5.38	0	Ser363, Asn382, Asn387 and Ser555	yes	[72]
5	SGTAV	5.54	0	Tyr334, Asn382, Ser383, Asn414, Arg415, Ser555 and Tyr572	yes	[72]
5	NSVAA	5.38	0	Ser363, Asn387, Asn414, Arg415, Ser508, Ser555 and Gly603	yes	[72]
4	DLEE	2.74	−3	Val418, Val465, Ile416, Arg415 and Val420	yes	[73]
5	LWNPR	10.73	+1	Ser363, Arg380, Asn382, Arg415, His436, Tyr572 and Phe577	yes	[74]
6	KPLCPP	9.29	+1	Arg380, Arg415, Gln530, Tyr525, Ala556 and Ser602	yes	[74]
8	YSNQNGRF	9.69	+1	Tyr334, Arg380, Asn382, Arg415, Ser508, Tyr525, Gln530, Ser555 and Ser602	no	[75]
3	SPW	5.42	0	Arg380, Asn382 and Ser 602	no	[76]
3	STW	5.42	0	Arg380 and Asn382	no	[76]
3	QKW	9.98	+1	Arg380, Asn387, Asp389, Arg415, Ser431 and Gly433	no	[76]
3	MKW	9.98	+1	Tyr525, Gln530 and Ser555	no	[76]
3	ETW	3.09	−1	Tyr334, Arg380 and Asn382	no	[76]
3	SVW	5.42	0	Tyr334, Arg336 and asn382	no	[76]
3	CNW	4.94	0	Gln528, Gln530 and Ser555	no	[76]
3	DHW	4.98	−1	Ser363, Arg380, Asn382 and Arg415	no	[76]
3	GQW	5.55	0	Gly480, Arg483, Arg415 and Ser508	no	[76]
3	SQW	5.42	0	Arg380, Asn382 and Tyr572	no	[76]
4	EGCG	3.09	−1	Asn414, Arg389, Ser 555 and Ser602	yes	[76]
3	VPN	5.4	0	Tyr334, Ser363, Asn382 and Gln530	no	[77]
4	DREL	4.0	−1	Arg135 and Gly148	no	[77]
3	DKK	9.63	+1	Arg380 and Asn382	no	[78]
3	DDW	2.78	−2	Arg415, Arg380, Asn382, Ser508 and Arg483	no	[78]
5	LYSPH	7.65	0	Tyr334, Ser363, Arg380, Asn382, Arg415, Arg483, Ser508, Tyr525, Gln530, Ala556, Tyr572, Phe577 and Ser602	no	[78]
6	LPHFNS	7.63	0	Tyr334, Ser363, Arg380, Asn382, Asn414, Arg415, Arg483, Tyr525, Gln530, Ser555, Ala556, Tyr572, Phe577 and Ser602	no	[78]
7	AEHGSLH	6.05	−1	Tyr334, Arg336, Ser363, Arg380, Asn382, Ser383, Pro384, Arg415, Ile461, Arg483, Ser508, Tyr525, Gln530, Ala556, Tyr572 and Ser602	no	[78]
7	FGPEMEQ	2.97	−2	Tyr334, Ser363, Arg380, Asn382, Asn387, Asp389, Asn414, Arg415, Gly433, Ile461, Ser555, Ala556, Tyr572 and Phe577	no	[78]
9	PSYLNTPLL	5.22	0	Tyr334, Ser363, Gly364, Arg380, Asn382, Arg415, Arg483, Tyr525, Gln530, Ser555, Ala556, Tyr572 and Phe577	no	[78]
3	DDL	2.91	−2	Ala366, Gly367, Arg415, Val465, Val512, Ile559 and Val604	yes	[79]
4	LSEE	3.09	−2	Ala366, Gly367, Arg415, Ile416, Gly462, Arg483, Gly509, Val512 and Val604	yes	[79]
4	TGEV	3.27	−1	Gly367, Arg415, Val418, Gly462, Leu557 and Val604	yes	[79]
4	TVEE	3.09	−2	Leu365, Val420, val514, Leu557, Ile559 and Val604	yes	[79]
4	TVET	3.27	−1	Leu365, Ala366, Arg415, Val418, Val465, Ile 559 and Val604	yes	[79]
4	TFEE	3.09	−2	Ala366, Arg380, Arg415, Val418, Ala510, Val512, Leu557 and Ile559	yes	[79]
4	LEHL	5.11	−1	Arg415, Val418, Val465, Val512, Leu557 and Ile559	yes	[79]
4	HELE	4.27	−2	Gly367, Arg380, Leu557, Leu559 and Val604	yes	[79]
5	NEGPQ	3.27	−1	Leu365, Arg380, Arg415, Val418, Val465, Val512 and Ile559	yes	[79]
7	WGDAGAE	3.01	−2	Gly367, Arg415, Ile416, Arg483, Val512, Ile559 and Val604	yes	[79]
4	ICRD	6.09	0	Tyr85, Ala 88, His129, Lys131, Val132, Arg135, Cys151, His154 and Val 155	yes	[80]
5	LCGEC	3.20	−1	His129, Lys131, Val132, Arg135, Met147, Gly148, Lys150, Cys151, His154 and Val 155	yes	[80]
6	RVIEPR	10.58	+1	Val369, Val467 and Val561	yes	[81]
7	SGFSTEL	3.13	−1	Val465, Ile559 and Val608	yes	[81]
7	ISREEAQ	4.09	−1	Gly367, Val418, Val465, Val467, Val512, Thr560, Val561, Val606 and Val608	yes	[81]
9	ERYQEQGYQ	4.08	−1	Gly372, Arg470, Val514, Ile559, Thr560 and Val608	yes	[81]
9	ERYQEQGYQ	4.08	−1	Gly325, Val369, Gly371, Gly372, Gly423 and Gln563	yes	[81]
11	LQEQEQGQVQS	3.03	−2	Gly325, Val369, Gly371, Val420, Val467, Val514, Thr560 and Met610	yes	[81]
11	KEEQTQAYLPT	4.08	−1	Gly325, Arg470, Ile559, Val561 and Val606	yes	[81]
13	IDNPNRADTYNPR	6.56	0	Gly371, Gly372, Gly423, Val514 and Gly564	yes	[81]
13	IDNPQSSDIFNPH	3.92	−2	Ser363, Asn382, Ser508 and Gly509	yes	[81]
14	NIDNPQSSDIFNPH	3.91	−2	Val369, Val420, Asp422, Gly423, Val467, Arg470, Val514, Thr560, Gln563 and Gly564	yes	[81]
6	SGFDAE	3.01	−2	Ser363, Leu365, Asn414, Ala510, Ser555, Ala556, Tyr572, Phe577 and Ser602	yes	[82]
8	YPFPGPIH	7.83	0	Arg 415 and Gly 367	yes	[83]
9	VTSALVGPR	11.6	1	Gly423, Val420 and Asn469	yes	[84]
10	DEQIPSHPPR	5.1	−1	Tyr334, Ser363, Arg380, Arg382, Arg415, Ser431, Gly433, His436, Gly462, Phe478, Arg483, Ser508, Gly509, Tyr525, Leu557 and Ser602	yes	[85]

A deep understanding of the structural basis of the Keap1-Nrf2 interaction aided in comprehending the most common characteristics of the bioactive peptides leads to a stable interaction with Keap1-Kelch domain. Keap1 dimerizes via its N-terminal BTB domain and attaches to one molecule of Nrf2 as a homodimer with two Kelch domains. The main interactions inside Nrf2 are two sequence motives at the N-terminus, the ETGE and the DLG motif. The ETGE motif has a roughly 100-fold stronger affinity for the Keap1-Kelch domain than the DLG motif. High-resolution X-ray structures of the Keap1-Kelch domain alone and in complexes with peptides containing the sequences of either the ETGE or the DLG motif of Nrf2 reveal the molecular details of the Keap1–Nrf2 interaction [64,65].

**Figure 4:**
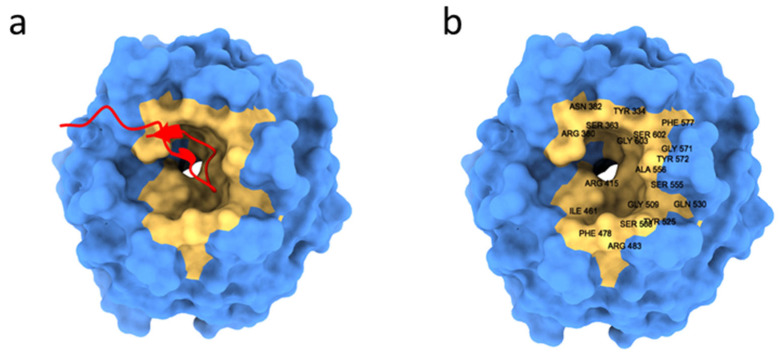
(**a**) Representation of the 2flu structure of the Keap1-Kelch domain (blue, PDB ID: 2FLU) in complex with the Nrf2 peptide (red). (**b**) The amino acids belonging to the five binding pocket subpackets are labeled highlighted in orange.

In summary, the Kelch domain is made up of six four-stranded antiparallel b sheets that combine to produce a highly symmetric six-bladed b-propeller. The b-propeller is a disc-shaped secondary structure that is roughly 36 Å in height and 49 Å in diameter. The ETGE and DLG binding motives of Nrf2 engage with a mostly positively charged bowl-shaped pocket extending at the bottom end of the disc. Peptides that bind to the Keap1-Kelch domain produce an extended structure, similar to a b-hairpin in the case of the ETGE peptide or a more complex helix shape in the case of the DLG motif Figure 4a [86].

The expanded structure’s tip, where the primary binding motif is found, extends into the bowl-shaped binding cavity of Keap1.

The Neh2 domain of Nrf2 can interact with the Keap1 binding site in the Kelch domain to induce its ubiquitination. The Keap1 binding pocket can be divided into five subpackets: P1 to P5: P1 (Arg415, Ile461, Gly423, Phe478, Arg483 and Ser508), P2 (Ser363, Arg380 and Asn382), P3 (Gly509, Ser555, Ala556, Gly571, Ser602 and Gly603), P3 (Gly509, Ser555, Ala556, Gly571, Ser602 and Gly603), P4 (Tyr525, Gln530 and Tyr572) and P5 (Tyr334 and Phe577) (Figure 4b) [86,87,88].

Salt bridges between negatively charged amino acids in the ETGE and DLG motifs and positively charged arginine residues of the Keap1-Kelch domain are crucial components of the interactions between Nrf2 and Keap1 [89]. For example, Glu79 of Nrf2 interacts with Arg415 and Arg483 of the Keap1-Kelch domain, while Glu82 of Nrf2 forms a second salt bridge with Arg380 in the case of the high-affinity ETGE motif of Nrf2.

The Keap1-Kelch domain and Asp29 of Nrf2 form a single salt bridge thanks to the low-affinity DLG sequence. Other weaker electrostatic contacts are also created, such as those between Gln26 and Asp27. The Nrf2 and Keap1 binding is primarily driven by a change in enthalpy, according to the thermodynamic parameters [90]. This is in favor of electrostatic interactions playing a significant role. The hydrophobic portion of the Keap1-Kelch domain, which is made up of, for example, Phe577, Tyr334 and Tyr572, is also contacted by peptides with the sequences of either the ETGE or the DLG motif [66].

The importance of salt bridges for the interaction between Nrf2 and Keap1 hampers the search for cellular active small-molecule inhibitors of protein–protein interactions. This makes it challenging to identify potent tiny molecules and shows that polar groups must be added to small molecules to counteract these intense ionic interactions.

Unfortunately, this may lessen the tiny molecules’ cellular permeability but it appears not to be a limitation in the context of binding peptides, as shown by the large variety of peptides that are able to bind to Keap1 (Table 1).

**Figure 5:**
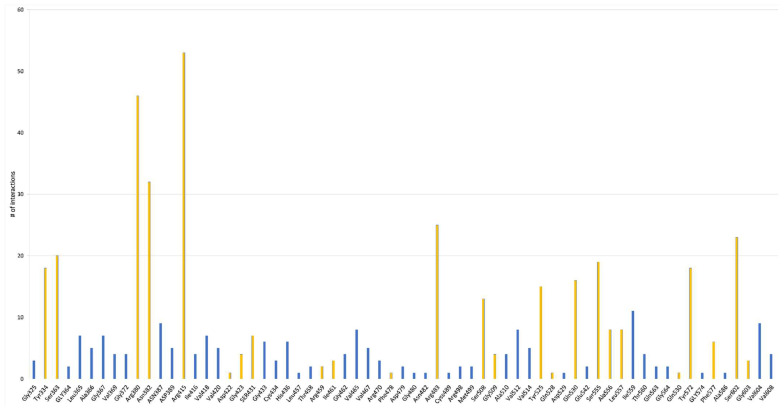
Interaction distribution between the docked antioxidant peptides (presented in Table 1 and Table S1) and the amino acids of the Keap1-Kelch domain. Amino acid belonging to the five binding pocket subpackets are labeled in yellow.

Considering how the different peptides interact with the Kelch domain of Keap1 (Figure 5, Table 1 and Table S1), it is noticeable that most frequent amino acids are the same when involved in the binding of Nrf2. Most of the interaction occurs with polar or charged amino acids, in particular, Arg (Arg 380, Arg 415 and Arg 483) and Asn (Asn 382), and secondarily with Tyr (Tyr 334, Tyr 525 and Tyr 572), Ser (Ser 363, Ser 508, Ser 555 and Ser 602) and Gln (Gln 530). Ile (Ile 559) is the only hydrophobic amino acid that is significantly involved in peptide binding. Taking into consideration the single peptide sequence, it was suggested that Pro, Gly, Ala, Val, and Leu are generally an essential presence in antioxidant peptides [67,68,69,91]. However, it has been discovered that a vast number of peptides with a heterogenous composition can bind to Keap1. In particular, peptides that show Glu residues, which engage electrostatically with Arg 380, Arg 415, Arg 483 and Asp residues, interact intramolecularly with Arg 415 to stabilize the hairpin conformation of the structure [61,62,70].

Moreover, the Glu residue of the soft-shelled turtle peptide EDYGA can directly bind to the Arg415 residue on the Kelch domain of Keap1 and create a hydrogen bond [61]. Similar to this, Asn382, Arg380 and Tyr334 on the Kelch domain of Keap1 might establish hydrogen bonds with the Glu residues in an amino acid sequence of RDPEER from a watermelon seed [71].

In addition, Ser residues in the amino acid sequence of the fermented milk protein APSFSDIPNPIGSENSE could create a hydrogen bond with the Arg415 and Ser363 residues on the Kelch domain of Keap1, and this peptide was experimentally proved to activate the Nrf2 pathway [63,68]. The importance of Ser residues was also recently shown by Huang P. et al. (2023) where all the Ser contained in the peptides SPSSS, SGTAV, TGVAS, GGSIT and NSVAA from pearl shell meat hydrolysate formed hydrogen bonds with the active site of Keap1 [72].

Therefore, although the recent literature shows that Pro, Gly, Ala, Val, Leu Glu and Ser are mainly involved in the binding with the Kelch domain of Keap1, it is not self-evident to identify which amino acid sequence could occupy the active site of Nrf2 as further investigations could easily enlarge the list of eligible candidates.

## 4. From In Silico Analysis to Cellular Studies

Putting together the information described so far, in the last few years some results confirming in silico evaluations of antioxidant bioactive peptides by cellular studies and vice versa are emerging. For example, in our previous work, in which the role of the milk bioactive peptides in the modulation of intracellular redox signaling was evaluated, molecular docking analysis was performed in order to confirm the results obtained in the cellular model [60,62,63]. Firstly, the peptides were shown to be cytoprotective from oxidative stress induced by H_2_O_2_ and TbOOH [63,92]. Secondly, their antioxidant activity was also confirmed by their ability to prevent ROS formation and lipid peroxidation. In more detail, a great decrease in ROS and TBARS levels was observed in cells pre-treated with the peptides, in particular with KVLPVPEK (K-8-K) and NTVPAKSCQAQPTTM (N-15-M), with respect to cells treated only with TbOOH. Additionally, the peptides increased the activities of antioxidant and phase II detoxifying enzymes suggesting that these compounds could have an effect on the regulators of cellular redox homeostasis. For this reason, Keap1/Nrf2 pathway activation was investigated in Caco-2 cells. As a result, K-8-K, N-15-M and QGPIVLNPWDQVKR (Q-14-R) were able to stimulate the translocation of Nrf2 from the cytosol to the nucleus, suggesting the activation of the Keap1/Nrf2 pathway. Molecular docking analysis showed that the antioxidant activity exerted by K-8-K, N-15-M and Q-14-R was due to the disruption of the interaction of Keap1 with Nrf2, since the peptides can affect the Nrf2 binding site of Keap1. A crosstalk between Nrf2 and NF-κB, mediated by the action of heme oxygenase (HO-1), one of the Nrf2-ARE regulated enzymes, is well-known. For this reason, the possible anti-inflammatory effects of antioxidant peptides in human cells were further explored [93]. Interestingly, with respect to the cells treated with TNF-α alone, K-8-K showed a decrease in the nuclear NF-κB levels and an expression of pro-inflammatory cytokines [93].

Similarly to what was highlighted in the studies reported above, Xing L. et al. (2021) observed that the dry-cured ham-derived peptide DLEE protected from ROS production and that some Nrf2-ARE regulated enzymes (GR, CAT and Gpx) increased their activity after the treatment of Caco-2 cells with the peptide [73]. The authors observed that the Nrf2 and Keap1 expression changed when the cells were treated with the DLEE. In order to confirm these results, a molecular docking analysis was performed. The DLEEs most active binding site was located in the central active pocket of the Kelch domain, involving Val418, Val465, Ile416, Arg415 and Val420 [73].

Accordingly, Zhu L. et al. (2022) extracted and identified peptides from fresh and hatched eggshells [74]. These authors observed that two peptides, KPLCPP and LWNPR, by hindering the Nrf2-binding site in the Keap1-Kelch domain, activated the cellular Keap1/Nrf2 signaling pathway by preventing the Keap1/Nrf2 interaction. This event can explain the cytoprotective action against oxidative stress in RAW264.7 cells.

Likewise, in a very recent paper, Huang P. et al. (2023) reported the antioxidant effects on HepG2 cells of six bioactive peptides identified from pearl shell meat hydrolysate, SPSSS, SGTAV, TGVAS, GGSIT, NSVAA and GGSLT [72]. Firstly, these peptides exerted a protective action on the cell viability in HepG2 cells treated with 2,2′-azobis (2-methylpropionamidine) dihydrochloride (AAPH) in order to induce oxidative stress. In particular, SPSSS, SGTAV and NSVAA increased SOD and CAT activities and Nrf2-ARE regulated antioxidant enzymes, reaching the levels of non-treated control cells. The authors showed that these peptides can interact with Tyr334, Ser363, Asn382, Ser383, Asn387, Arg415, Tyr572 and Phe577 in the active site of Keap1 [72].

In another work regarding gastrointestinal digestion simulation of fermented soy, molecular docking analysis was utilized as a selection strategy in order to identify the possible antioxidant peptides [62]. Peptides were extracted and identified from the digested fermented soy. Subsequently, in order to select possible antioxidant peptides that can activate the Keap1/Nrf2 pathway, a molecular docking analysis with Keap1 of the newly identified peptides was carried out. Finally, the effects in Caco-2 cells of the selected peptides on the Keap1/Nrf2 pathway were investigated in order to elucidate the mechanism of action of these soy-derived peptides. DEQIPSHPPR (D-10-R) and SLVNNDDRDS (S-10-S) exerted protective action in Caco-2 cells against ROS production and changed the expression of antioxidant and phase II enzymes. Moreover, D-10-R and S-10-S increased the nuclear Nrf2 levels in the cells. The results obtained in the cellular model were consistent with the molecular docking data as the most antioxidant peptides were those showing the highest interaction score with the Keap1-Kelch domain [62].

Moreover, a molecular docking analysis is now considered as part of the screening strategy in order to understand if new peptides can exert antioxidant effects [75,76].

A molecular docking analysis is also used in order to evaluate the possible anti-hypertensive effects of the bioactive peptides confirming the importance of this method in the selection process of these molecules [94,95].

The experimentation reported above was carried out in cellular models in vitro; however, Jiang Y. et al. (2021) described similar results obtained in AAPH-treated Sprague Dawley (SD) rats. The induced oxidative stress allowed the assessment of the antioxidant bioactive peptide’s effects in vivo [77]. In particular, the authors tested two peptides, VPN and DREL, which were extracted from Jiuzao (the residue that remains after the distillation of a famous alcoholic Chinese beverage, Baijiu). These two peptides showed a great protective effect against oxidative damage induced by AAPH in rat organs. Moreover, antioxidant enzyme activities and GSH levels increased, while GSSG and MDA levels decreased, both in the serum and tissues of the rats treated with VPN and DREL. The two peptides upregulated Nrf2, MafK, p38 and PI3K, with respect to the AAPH-alone group. The results were supported by a molecular docking analysis, which showed that VNP interacts with Tyr334, Ser363, Asn382, Gln530 and Ser602, while DREL interacts with Arg135 and Gly148 of the Keap1-Kelch domain. DREL also exhibited anti-inflammatory properties through the activation of Nrf2/HO-1 axis, inhibiting the release of TNF-α, IL-1β, IL-6 and NO [77].

Clearly, there is a close correspondence between the in vitro and in vivo results.

## 5. Conclusions

From the data discussed above, it is evident that peptides that are able to interact with the Keap1-Kelch domain share some common features. The correlation between the structural in silico prediction and the functional evaluation of the antioxidant bioactive peptides with their molecular mechanisms gave fruitful results. In fact, it was observed that the presence of proline residues in the peptide sequences is critical for folding and endows the peptides with a strictly specific conformation that is able to promote the binding of these compounds into the Keap1 pocket. Moreover, the presence of charged amino acidic residues, such as Lys, Gln, Asn and Arg at the N- and C-terminal side of the molecules, exerts a crucial role for the interaction with the Keap1 pocket, as most of the interaction occurs with polar or charged amino acids of the Keap1-Kelch domain.

Concluding, the molecular docking approach is proposed as a useful tool in order to select new antioxidant bioactive peptides interacting with Keap1.

**Figure 6:**
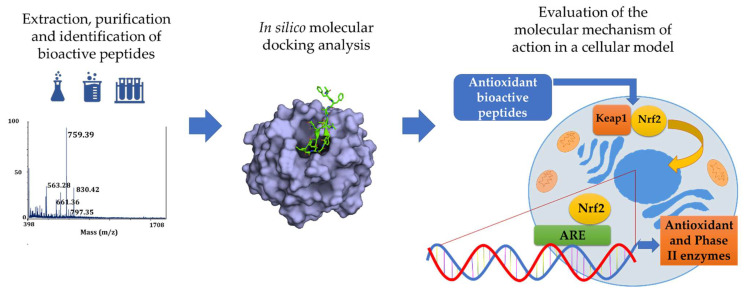
Proof of concept and conclusions.

The identification of these specific properties of the peptide sequences could be useful for the development and rational design of drugs that target the Keap1/Nrf2 pathway, regulating redox homeostasis. Considering that an imbalance of the redox status of the cells is related to pathogenesis of the majority of diseases, understanding the role of specific molecules, such as food-derived peptides, on redox regulation can be of interest for devising new nutraceuticals or drugs. This innovative approach and the related considerations reported here (Figure 6) may be useful for other researchers, giving an integrated view of the problem and explaining how an antioxidant molecule can act.

## Data Availability

The analyzed data are reported in Supplementary Materials.

## Supplementary Materials

The following supporting information can be downloaded at: https://www.mdpi.com/article/10.3390/antiox12030665/s1. Table S1: Interaction between the docked antioxidant peptides and Keap1-Kelch domain.
